# COVID-19 in Singapore and Malaysia: Rising to the Challenges of Orthopaedic Practice in an Evolving Pandemic

**DOI:** 10.5704/MOJ.2007.001

**Published:** 2020-04-07

**Authors:** K Tay, T Kamarul, YL Woo, M Mansor, X Li, J Wong, A Saw

**Affiliations:** 1 Department of Orthopaedic Surgery, Singapore General Hospital, Singapore; 2 Department of Orthopaedic Surgery, University Malaya Medical Centre, Kuala Lumpur, Malaysia; 3 Department of Anaesthesiology, University Malaya Medical Centre, Kuala Lumpur, Malaysia; 4 Department of Obstetrics and Gynaecology, KK Women’s and Children Hospital, Singapore; 5 Division of Anaesthesiology, Singapore General Hospital, Singapore

**Keywords:** epidemic, public health, emergency, coronavirus, social distancing

## Abstract

With the increasing number of COVID-19 cases and related deaths worldwide, we decided to share the development of this condition in Singapore and Malaysia. First few cases were diagnosed in the two countries at the end of January 2020, and the numbers have surged to thousands by end of March 2020. We will focus on strategies adopted by the government and also the Orthopaedic community of the two countries up till the beginning of April 2020. We hope that by sharing of relevant information and knowledge on how we are managing the COVID-19 condition, we can help other communities, and health care workers to more effectively overcome this pandemic.

## Introduction

On 31st Dec 2019, the World Health Organization (WHO) China office was informed of a type of respiratory infection in the city of Wuhan which was noted to be different from previously known coronavirus infections that causes SARS (SARS-CoV) and MERS (MERS-CoV)^1^. This novel virus (SARS-nCoV) was later re-named Severe Acute Respiratory Syndrome coronavirus two (SARS-Cov-2), and the genomic sequence was published on 7th January 2020. With the development of the reverse transcription polymerase chain reaction (RT-PCR) test kit to detect the presence of SARS-CoV-2, confirmation of the disease (COVID-19) can be made^2^. Within the following weeks, new cases were detected in Thailand, South Korea, and Japan, and their epidemiological patterns started to indicate that human to human transmission is possible^3, 4^. Considering the mortality and morbidity risk of the national population, the Chinese government decided to implement lockdown of the province of Hubei on 23rd January 2020, one day before the Spring festival celebration where millions of people were expected to travel across the whole country^5^. On 30th January 2020, with new confirmed cases in United States, Europe, Middle East and Australia, WHO declared the condition to be a Public Health Emergency of International Concern (PHEIC)^6^. By means of early confirmation of new cases, stringent contact tracing, social distancing and travel restrictions, the number of new COVID-19 cases seems to have plateaued in South Korea and Japan, and even reduced in China in the month of March. However, with more than 110,000 cases from 114 countries reported globally, WHO declared the status of pandemic on 11th March 20207.

### Early Response

**Fig. 1:**
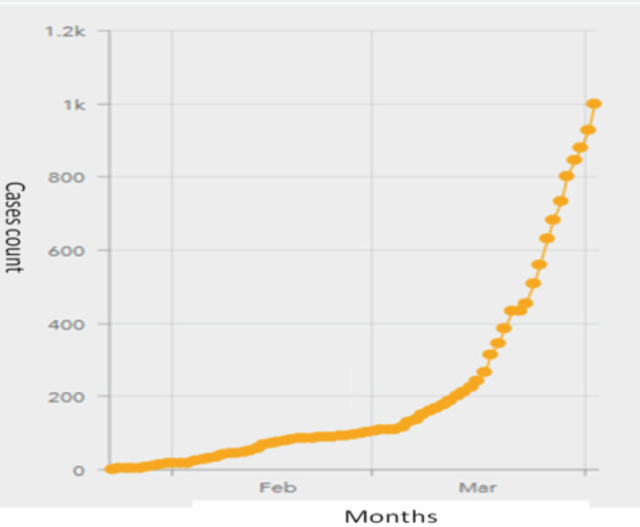
No of COVID-19 cases in Singapore by date^10^.

Singapore, a city state in Southeast Asia with a highly robust travel network within the region and East Asia, rapidly became the epicentre of the COVID-19 epidemic in late January to early February, with several countries imposing travel restrictions to Singapore^8^. The index imported COVID-19 case was diagnosed in Singapore on 23rd January 2020. Highly pro-active measures were immediately taken in a coordinated multi-ministry taskforce set up by the Singapore government in close cooperation with the country’s healthcare services to try to contain the impact of the epidemic^9^. In spite of this, spikes were still observed which can be broadly described as 1) imported cases from mainland China, 2) local clusters, and 3) imported cases from returnees from other parts of the world (Fig. 1)^10^. In spite of a rigorous protocol for contact tracing, there remains a slight uptick in unlinked cases diagnosed locally^11, 12^.

**Fig. 2:**
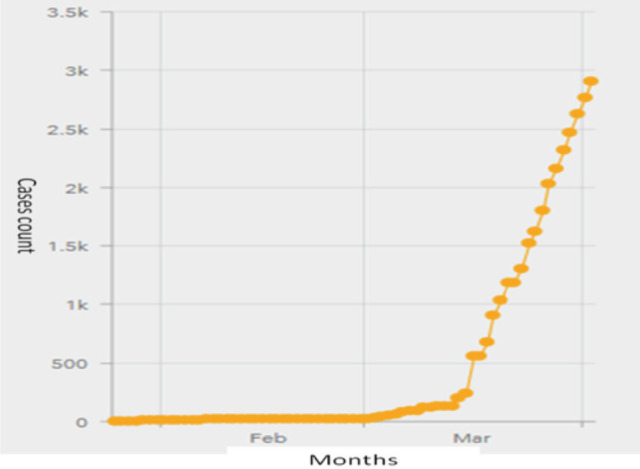
No. of COVID-19 cases in Malaysia by date^10^.

On 25th January 2020, three Chinese nationals touring Malaysia were diagnosed to have COVID-19. Thermal scanners were installed on all entries to the country, and on 27th January the government identified 26 hospitals to handle investigations and treatment of COVID-19 patients^12, 13^. On 30th January, a committee coordinated by the National Disaster Management Agency (NADMA) in collaboration with few other ministries was formed to organise a return of Malaysian nationals from Hubei, China. Up till end of February, most of the 25 confirmed cases in Malaysia were foreigners who had contacted the disease before they enter the country^13^. A second cluster of cases that was associated with a religious gathering in Petaling Jaya emerged in early March^14^. On 16th March, the Prime Minister announced two-week period of Movement Controlled Order (MCO) that involved closing of international borders, shutdown of non-essential businesses, prohibition of travelling and public gatherings^15^. However, confirmed number of cases surged to more than 2000 in the month of March (Fig. 2)^10^, and roughly half of the cases were associated with the second cluster^15^. MCO was later extended to 14th April^16^.

The Singapore National Centre for Infectious Diseases handles the majority of COVID-19 cases in the country while isolation facilities with capabilities for intensive care are also created in all public hospitals. As the workload increased, recovering COVID-19 patients are also housed in private hospital facilities within Mount Elizabeth Hospital and Gleneagles Hospital. Across the whole Malaysia, the Ministry of Health has identified 35 hospitals to serve as COVID-19 admitting hospitals, and the remaining hospitals including the health centres as screening centres^17^. With close to ten-fold increase of total number of confirmed cases in the month of March (from 25 to more than 2,000), all these hospitals have been procuring essential material including testing kits, personal protection equipment (PPE), medicines and ventilators^18^.

Although the Orthopaedic surgery specialty may not be directly involved in the care of COVID-19 patients with predominantly respiratory problems, we are actively participating in various aspects of the preparation. Beyond significant disruptions to daily supplies and manpower resources due to the traditionally high inter-dependence of Malaysia and Singapore, the sudden surge in healthcare resources diverted to the current pandemic, which looks increasingly to be protracted, is also challenging effective services for elective and chronic conditions in terms of:

Sudden reduction in inpatient beds and resources:

- Potential risk for healthcare transmission
- Overwhelming of the healthcare infrastructure leading to an inability to deal with other important health issues
- Lack of trained manpower in the event of staff morbidity and mortality from COVID-19
- Affected healthcare staff morale

### General Principles of Safety and Isolation in Orthopaedic Service

In preparation for a potentially drawn out fight against a pandemic, a robust comprehensive plan was crafted in order to maintain continuity of care for all surgical disciplines including Orthopaedic surgery. Workflows were created and refined through a series of in-situ simulations^19^. These encompassed coordination of staff, movement of surgical equipment, infection prevention practices and decontamination following the procedure. Orthopaedic teams performing surgeries would have to outline all the equipment they would require during such surgeries to facilitate the move of the necessary equipment to this standalone facility. This would require forward planning, and examples of orthopaedic cases requiring this workflow would be debridement for necrotising fasciitis in a suspect COVID-19 case^19^.

The simulations were performed by anaesthesia and surgical teams, using various scenarios with different surgical disciplines. These aided in refining such workflows and identifying possible problems, such as inadequacies in coordination and communication, environment limitations, unsatisfactory equipment set-up, and unfamiliarity with protective equipment and infection control measures. To address these, an anaesthetist would be appointed during every case to oversee and ensure that essential steps which might have compromised patient and staff safety were not missed^19^.

### A: Operating Theatre (OT)

Based on the principle of complete segregation and in accordance to “Disease Outbreak Response System Condition” (DORSCON) Orange^20^ directive to prevent potential cross contamination between healthcare workers, the operating theatre (OT) team was segregated into two separate sub-groups; two teams to deal with acute trauma cases and one team for urgent spine surgeries. This allowed the department to continue service provision for Orthopaedic cases in Singapore while adhering to strict infection control measures. For the general trauma OT teams, each team comprised of a complete subset of sub-specialty surgeons from tumour surgery, arthroplasty, foot and ankle surgery and pelvic and acetabular specialist^21^. Each team had sufficient expertise to handle complex cases that required specific skillsets. The spine team focused on operating on patients that required urgent decompression or restoration of spine stability. The trauma OTs and spine OT ran during office hours. The OT location was chosen to be at least two theatres apart to further ensure physical distancing and to prevent cross contamination

The surgical management of COVID-19 was carried out in Malaysia according to the 5th Edition Ministry of Health Guidelines For COVID-19 Management^22^ which was released on 25th of March 2020. A special core of COVID-19 orthopaedic team with at least one specialist, two medical officers and two staff nurses were identified to standby for all suspected COVID-19 patients, and should be optimally trained in managing COVID-19 patients including handling the PPE, sample taking and packaging.

**Fig. 3:**
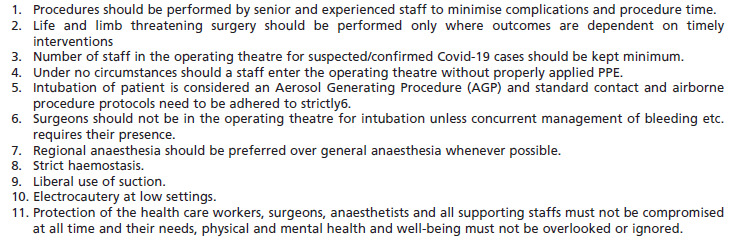
Intraoperative guidelines in managing COVID patients/PUI for the orthopaedic cases.

The most fully equipped, dedicated OTs for COVID-19 patients /Person under Investigation (PUI) were immediately made available. The OT chosen ideally is one with negative air pressure or one that will minimise OT contamination, staff exposure and nearest to the entry point. The non-COVID operation theatres were further divided into Emergency and Semi-Emergency OT. All Electives cases were being postponed indefinitely. Surgical management of emergencies such as fractures, dislocations, tumours and infections might still be undertaken and surgeons must adhere to the recommendations of the Surgical and Orthopaedic Association regarding this. The emergency and semi-emergency cases were required to be tested for COVID-19. Even if they were tested negative, the non-COVID cases would still be handled by health care workers (HCW) in full Personalised Protective Equipment (PPE). This is to protect the HCW against “false negative cases”^23, 24, 25^ (Fig. 3).

### B: Ward / Inpatient Management

The Ward/in-patient management team encompassed not only the management of patients directly managed under Orthopaedic Surgery, but also for inpatient referrals made by other specialties. The ward group was segregated into two main groups that of a “clean ward”, meant for patients without any infections or open wounds, and a “dirty ward”. The former had patients with spine conditions or closed fractures; there were no elective surgeries during this period; however, patients pending discharges after elective surgeries were kept in this ward. The latter ward was for patients with musculoskeletal infections, and the staff here were also the designated teams to round patients admitted to wards outside of these two wards (“overflow” wards), or suspected or confirmed COVID-19 cases. This was to ensure that should a team be inadvertently exposed to a suspected or confirmed case, rapid contact tracing and isolation of relevant staff could be performed. Each ward was also divided up by bed numbers into individual sectors. The sectors were covered by teams comprising consultants/associate consultants, registrars, and medical and house officers. This afforded a degree of geographic separation amongst doctors, and also ensured continuity of care with the same team of doctors covering an assigned number of patients. Across the two wards, surgeons from varying subspecialties were present, mirroring the admixture of surgeons in the operating theatre. In the lead up to the segregation, the Department ensured that patients fit for discharge were either discharged home or transferred to community hospitals to reduce the likelihood of exposure to COVID-19 of existing inpatients, to free up beds for admission of suspect COVID-19 cases and ensure manageable workload. Plans were laid down for discharge planning and management of ongoing medical issues were handed to the teams designated to cover relevant ward sectors. The ward group command team met on a regular basis to be updated regularly discuss Standard operating procedures (SOPs) perform pre-surgical audits, and discuss any critically ill, febrile or potential COVID-19 patients. The highest attention was paid to SOPs designed towards the management of potential or confirmed COVID-19 cases. These guided the doctors on the identification of potential cases in consult with the Department of Infectious Diseases, and minimise the number of staff potentially exposed for adequate patient care.

In Malaysia, the management of COVID orthopaedic ward or inpatient is based on the MOH guidelines for the management of COVID-19 patients. The COVID-19 patients /PUI were nursed in the infectious disease ward, unless patients required intensive or specialised orthopaedic attention. These patient will be nursed in an individual room or isolation room in the orthopaedic ward. The limited negative pressure rooms were reserved for those confirmed to be infected. Just like in Singapore, patients fit for discharge were either discharged home or transferred to other public or private hospitals to reduce the likelihood of exposure to COVID-19 of existing inpatients and to free up beds for admission of suspected COVID-19 cases. Each surgical unit is recommended to have their own management pathways based on their own logistics and resources.

### C: Outpatient Clinic Management

The usually heavy Outpatient clinic poses potential challenges in terms of potential disease clusters and inadvertent lapses due to work overload. Actions were taken to maintain a reasonable workload and minimise staff and patient contacts by:

- Cutting down non-essential workload such as patients on long term follow-up or conservative management^26^
- Limiting ourselves to urgent care such as trauma, infection, acute spine emergencies and tumour referrals
- Allowing for off-site prescription top-ups, extension of medical leave and correspondences for whatever indications

As the Outpatient services can be exposed to patients from various sources, a strict screening protocol in accordance to existing Singapore Ministry of Health guidelines covering travel histories, contact histories, and coryzal symptoms were taken before admittance to the Outpatient clinic. Cases deemed at-risk were seen if required for urgent conditions. These patients were immediately isolated, and consults and any outpatient procedures performed in full personal protective equipment (PPE). At-risk patients with non-urgent conditions had appointments deferred for three weeks as a safety precaution.

In Malaysia, face to face patient consultations appointments carry significant risk due to the COVID-19 pandemic, with the increasing incidence of community spread of the disease. In order to restrict face to face consultations, only the walk-in urgent cases are seen in the clinic. Patients will be strictly screened as above and triage. At the clinic, social distancing is practised at all times. Non-essential consultations such as for patients on stable long term follow-up and non-urgent cases will be minimised by offering them with longer duration of off-site prescriptions, offering telephone consultations and internet consultations where possible. Clinic nurses and support staff will contact patient listed for follow-up to offer them alternative follow up dates, and this would include postponement of scheduled blood or imaging investigations. Medical record office of the hospitals will facilitate easy access to digital or online medical records for the clinicians to evaluate the condition of selected patients to ensure those requiring early attention especially the Orthopaedic oncology patients will not be neglected. Consultation via online facilities was also available for new patient referrals, so that a management plan can be partially implemented for patients seeking an opinion and treatment.

### D: Trauma / Emergency Service

To cater for the surge in inpatient beds required for treating acute COVID-19 patients requiring hospitalisation, institutions in Singapore developed central command teams which reviewed overall healthcare resources available on a weekly basis.

The majority of elective surgeries were postponed, with allowances for urgent surgeries such as tumour, infective cases, acute and delayed trauma and spine surgeries with deteriorating neurological status. Inpatient beds were also created in community hospital facilities that are within close proximity of the surgical teams to cater for these surgeries^26^. When the government of Malaysia announced the MCO on 17th March 2020, all the admitting hospitals postponed their semi-elective Orthopaedic procedures and cut down semi-elective / trauma cases. Facilities for managing emergency cases like open fractures and polytrauma is maintained, but patients would be referred to other government hospitals once the conditions have been stabilised. Some private hospitals have also offered to share their services in managing trauma patients with discounted overall fee that is comparable to government hospitals. With the reduced road traffic and presence of enforcement officers along all major highways, we did not anticipate many road traffic accidents during this period.

### Medical Education, Orthopaedic Training During Covid-19

**Fig. 4:**
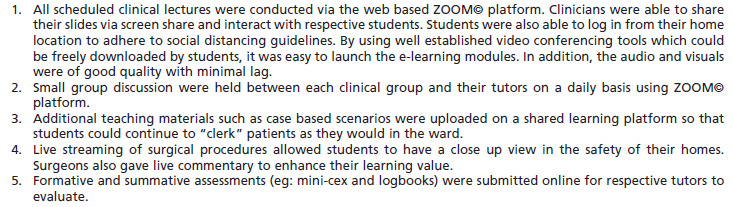
Online models of clinical teaching for medical undergraduates^26^.

Since Singapore escalated to DORSCON Orange in February 2020, all medical students were barred from clinical postings to reduce the risk of exposure and infection. With such short notice, medical educationists had to adapt quickly to modify the clinical curriculum without significantly impacting students’ learning. In order to do so, we harnessed the powers of technology to bring the entire clinical curriculum online^27^ (Fig. 4). Whilst clinical posting and actual patient interaction cannot be replaced by e-learning, in extenuating circumstances such as the current COVID-19 situation, online learning has proven to be effective and well received by both students and tutors. Importantly, this can avert an unnecessary delay in our medical students’ progression.

Similarly, undergraduate medical education in public and private medical schools in Malaysia have been temporarily modified to adopt online mainly teaching through webinars and videoconferencing. The impact would be worse for students who are preparing for upcoming final professional examination especially for institutions where clinical performance is an essential component for this evaluation. For postgraduate masters Orthopaedic training, rotation posting in the six teaching Universities will remain static during the CMO period in Malaysia. All general Orthopaedic and subspecialty courses, short-term fellowship postings in neighbouring countries, inter-hospital exchange postings, and department meetings have been temporarily cancelled. Following a recent meeting of the Malaysian Medical Deans’ Council, the national Orthopaedic Specialty Committee (OSC) part-2 examination which was supposed to be held end of April was postponed to November 2020. It is not advisable to request the candidates for the examination to travel, and nearly impossible to convince patients to volunteer as clinical case models for the clinical components of the examination. In addition, several regional and international conferences scheduled to be organised in the two countries for the next few months have also been postponed, and this includes the ASEAN Orthopaedic Association Congress in Kuala Lumpur^28^.

### Future Directions: Building Resilience and Infrastructure to deal with Future Pandemics

The COVID-19 pandemic is panning out to be a protracted situation. By practicing strict inter-team segregation both in actual working space and during rest times, we are able to ensure that only one team will be affected should any doctor be involved in a suspected or confirmed COVID-19 case. Ensuring staff safety is of paramount importance. A list of the appropriate PPE is drawn up and included in all staff briefings. Stocks of appropriate PPE are made available at all settings and readily accessible to clinical staff in the course of their daily duties for handling suspect cases. Training should be performed for mask-fitting and ensure staff comply with appropriate methods of donning themselves with the full PPE, especially in handling suspect or confirmed cases.

Communication is continuously maintained between the Inpatient/Ward, Operating theatre and Outpatient teams. To achieve proper hand over of cases and continuity of care, a pair of doctors comprising of one specialist and one trainee from the Operating theatre team is appointed as the point-of-contact (POC) to liaise with colleagues manning the ward using confidential communication tools such as TigerText. Similarly, the doctors in the clinic group communicate any concerns or instructions to the ward group for any patients admitted from clinic, and the ward group reciprocated for any patients requiring attention to specific concerns during outpatient reviews on discharge. At all times, suspected COVID-19 cases are highlighted, so that every transition between the clinic, ward and operating theatre is carefully managed to minimise exposure to staff.

The command team has also looked into issues of maintaining cohesion and morale amongst the junior staff, to avoid staff burn out. Working arrangements are made such that there is a degree of redundancy in the system, such that doctors have adequate rest days during the weekend. Seniors maintain vigilance to look for signs of any stress in junior staff, particularly when it comes to exposure to potential COVID-19 cases. In the local context this is needed, as junior doctors sometimes feel reticent to share any issues of stress or burn out, for fear of “letting their peers down”.

From the early stages of the COVID-19 epidemic in Wuhan, China, WHO has initiated research and development in diagnostics, vaccines and therapeutics for this infection. Although the natural history of the condition is still far from clear, there are many pertinent questions that need to be answered urgently^29^. In our search for treatment, cure and prevention for COVID-19, we have to rely on well-designed clinical trials^30^. One example would be the large multinational SOLIDARITY trial that is designed to test the effectiveness of four groups of drugs that have the potential to be used for COVID-19. Preventive measure like development of an effective vaccine would probably be months if not years away^31^. Although the field of research may not be directly related to Orthopaedic service, as a component of the medical profession, it is important for us to support and participate in these researches^32^. A survey of Orthopaedic surgeons in Wuhan who were diagnosed with COVID-19 is a good example of how we can contribute towards better understanding of this condition^33^.

Having a shared border between Singapore and Malaysia entails potential challenges after lifting of the MCO with resumption of human traffic across the borders. Though it entails some risk of cross border transmission, effective precautions would include:

- Enforcing the Stay Home Notice (SHN) or Movement Control Order (MCO) to reduce non-essential travelling of the public.
- Rigorous screening and self-monitoring measures
- Appropriate social distancing

Continuous data sharing and joint policy-making processes between medical personnel from both countries. This follows on a Joint COVID-19 Taskforce between Malaysia and Singapore already in place^34^.

## Summary

The COVID-19 pandemic has caught many countries off-guard though regions previously exposed to the 2003 SARS-CoV outbreak are somewhat better prepared. With more than 600 million population over a relatively small geographical region, South East Asia will have to be better prepared for public health emergencies of this nature Table I^10^. Various medical specialties should utilise all available channels of communication to share our experience, learn from each other, and join force with our partners from other parts of the world to overcome this challenge. We hope that through this sharing of information between Orthopaedic communities in this region, we can generate more interest among our medical fraternity to collaborate and join forces to overcome this pandemic.

**Table I:** Number of COVID-19 cases in ASEAN countries and cumulative no of related deaths^10^

	Cumulative no. of confirmed cases				No. of deaths
	1-Jan-2020	1-Feb-2020	1st Mar 2020	3-Apr-2020	3-April 2020
Malaysia	0	3	24	2,908	45
Philippines	0	2	3	2,311	205
Thailand	0	19	43	1,875	15
Indonesia	0	0	2	1,790	170
Singapore	0	3	102	1,049	4
Vietnam	0	6	16	222	0
Brunei	0	0	0	131	1
Cambodia	0	1	0	109	0
Myanmar	0	0	0	16	1
Lao	0	0	0	10	0

## Dedication

We would like to dedicate the article to Dr Lukman Shebubakar from Jakarta, who passed away on 4th April 2020 due to COVID-19 infection. His contribution towards medical service and Orthopaedic training in Indonesia and this region will always be remembered.
